# Genome-scale metabolic modelling of the human gut microbiome reveals changes in the glyoxylate and dicarboxylate metabolism in metabolic disorders

**DOI:** 10.1016/j.isci.2022.104513

**Published:** 2022-06-02

**Authors:** Ceri Proffitt, Gholamreza Bidkhori, Sunjae Lee, Abdellah Tebani, Adil Mardinoglu, Mathias Uhlen, David L. Moyes, Saeed Shoaie

**Affiliations:** 1 Centre for Host–Microbiome Interactions, Faculty of Dentistry, Oral & Craniofacial Sciences, King’s College London, London, SE1 9RT, UK; 2 Science for Life Laboratory, KTH–Royal Institute of Technology, Stockholm, Sweden

**Keywords:** Microbiome, Systems biology, Metabolomics, Omics

## Abstract

The human gut microbiome has been associated with metabolic disorders including obesity, type 2 diabetes, and atherosclerosis. Understanding the contribution of microbiome metabolic changes is important for elucidating the role of gut bacteria in regulating metabolism. We used available metagenomics data from these metabolic disorders, together with genome-scale metabolic modeling of key bacteria in the individual and community-level to investigate the mechanistic role of the gut microbiome in metabolic diseases. Modeling predicted increased levels of glutamate consumption along with the production of ammonia, arginine, and proline in gut bacteria common across the disorders. Abundance profiles and network-dependent analysis identified the enrichment of tartrate dehydrogenase in the disorders. Moreover, independent plasma metabolite levels showed associations between metabolites including proline and tyrosine and an increased tartrate metabolism in healthy obese individuals. We, therefore, propose that an increased tartrate metabolism could be a significant mediator of the microbiome metabolic changes in metabolic disorders.

## Highlights

• Metagenomic analysis highlights key common bacterial species across metabolic diseases
• Metabolic models showed higher levels of acetate produced by disease enriched bacteria
• Reaction analysis revealed increases in the glyoxylate and dicarboxylate pathway
• Metabolomics and modeling analysis showed the potential role of tartrate metabolism

## Graphical abstract

**Figure:**
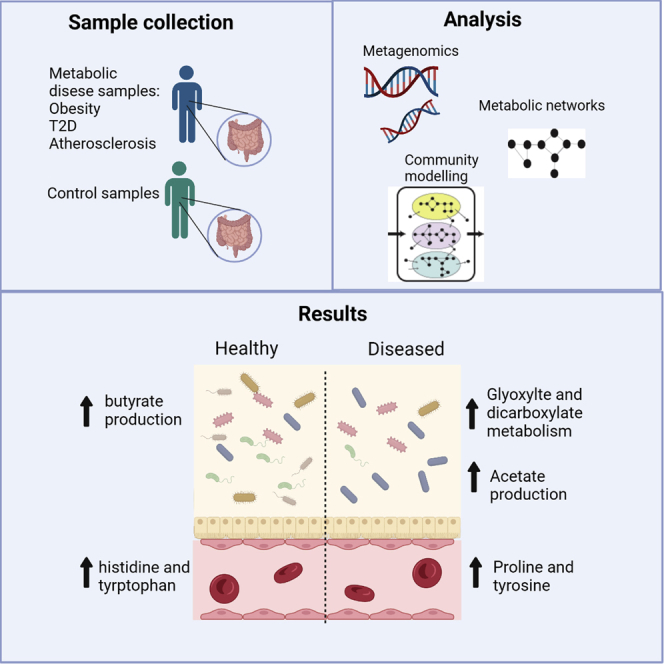

## Teaser

Microbiome; Systems biology; Metabolomics; Omics; Metabolomics

## Introduction

In recent years we have seen significant advances in elucidating the importance of the gut microbiome in human health and disease (Gentile and Weir, 2018; Zomorrodi and Maranas, 2012). Microbial communities have an intimate symbiotic relationship with their host, promoting protection against pathogenic microbes, maintenance of homeostasis, and processing of nutrients otherwise indigestible by humans (Zhang et al., 2019). Shifts and alterations in the microbiome have been linked to different environmental factors including age, geography, and body mass index (BMI) (Le Chatelier et al., 2013b; Yatsunenko et al., 2012). In addition, diet has a significant impact on the microbiome (David et al., 2014). Various diseases have been associated with altered microbiome composition, and many studies have shown these communities can contribute to metabolic inflammation and metabolic disorders (Fan and Pedersen, 2021; Johnson et al., 2017; Proffitt et al., 2020; Tilg et al., 2020). There are several studies linking major metabolic disorders (type 2 diabetes (T2D), obesity, and atherosclerosis cardiovascular disease (ACVD)) with changes in gut microbiota composition (Karlsson et al., 2012, 2013a; Le Chatelier et al., 2013b). These dysbiotic changes resulting from a loss in stability of the gut microbiome drive changes in the gut ecosystem that result in a reduction of microbial diversity (Litvak et al., 2018). In turn, this results in shifts in key metabolite production. For example, short-chain fatty acids (SCFAs) such as acetate regulate immune cell production and help maintain intestinal homeostasis (Yang et al., 2020) and have been linked with promoting obesity and insulin resistance (Perry et al., 2016). Likewise, amino acid (AA) metabolism in the gut microbiome has been observed to have a large impact on health and disease (Cook and Sellin, 1998; Oluwagbemigun et al., 2020). In particular, plasma glutamate levels positively correlate with increased BMI and with fasting triglycerides, both of which can lead to insulin resistance (Palomo-Buitrago et al., 2019).

Despite a large number of gut metagenomic studies in metabolic diseases, the contribution of specific microbes to host metabolism during metabolic disease, systematic analysis, and modeling has not been mechanistically studied. In particular, little is known about the specific impact of microbe-microbe metabolic interactions. Recent advances in the metabolic modeling and reconstruction of genome-scale metabolic models (GEMs) have enabled the study of species-specific metabolisms and metabolic interactions within microbial communities (Gu et al., 2019; Magnusdottir et al., 2017; Shoaie et al., 2015). These computational models provide a platform that can be used to explore the genotype-phenotype relationships thereby allowing us to predict different phenotypic possibilities for microbes under different sets of constraints (Karlsson et al., 2011). This facilitates the study of interactions between species, and the quantification of growth, consumption, and production of metabolites. Using constraint-based modeling, diverse phenotypes under different conditions, such as nutrient availability can be simulated (Bordbar et al., 2014).

In this study, we focused on gut microbial metabolic modeling of three main metabolic disorders, ACVD (Jie et al., 2017; Karlsson et al., 2012), obesity (Le Chatelier et al., 2013b; Qin et al., 2010), and T2D (Karlsson et al., 2013a; Qin et al., 2012a). We used publicly available gut metagenomics datasets from these cohorts along with a previously generated list of significantly associated species in each study from our recent human gut microbiome atlas; Database: www.microbiomeatlas.com (Shoaie et al., 2021). These datasets were previously analyzed based on the recent gut integrated gene catalog (Wen et al., 2017), and the metagenome species profiled using our recently updated 1,989 Metagenomic Species Pan-genomes (MSPs) (Plaza Oñate et al., 2018). We applied GEMs for these species to investigate how gut microbiome metabolism varies between these three metabolic disorders. Individual and community-level modeling were performed together with reaction abundance to pinpoint specific bacterial metabolites and reactions associated with each metabolic disorder. Our results provide insights into the mechanistic role of gut microbiome in metabolic diseases and how this role is potentially similar between these three pathologies. Our models predicted the results of previous studies such as an increase in acetate (Schwiertz et al., 2010) and depletion of butyrate levels (Arora and Bäckhed, 2016). In addition, our modeling indicated a disparity in the production of amino acids including glutamate, proline, tyrosine, and valine in the gut microbiome between the healthy and disease cohorts. Performing reaction abundance analysis between cohorts demonstrated multiple reactions involving tartrate metabolism which were enriched in all three disorders. We performed personalized community modeling analysis for two of the cohorts, leading to further clarification of bacterial metabolism differences between healthy and diseased subjects. These findings were further evaluated by investigating the link between the tartrate metabolism and circulating amino acid levels using the host blood metabolites thus showing the potential impact of the tartrate on the host metabolism.

## Results

To investigate the individual metabolic roles of the microbiome in metabolic disorders of the host, we used gut metagenomics data from six previous studies on obesity, T2D, and ACVD (Jie et al., 2017; Karlsson et al., 2012, 2013b; Le Chatelier et al., 2013b; Qin et al., 2010, 2012a). This amounted to 1,443 subjects in total; 278 obese patients and 263 matched controls; 271 patients with T2D and 231 matched controls; and 219 patients with ACVD and 181 matched control samples (Tables 1 and 2). Previously, we have analyzed these data with an updated gut gene catalog and MSP profile (Shoaie et al., 2021) while here we performed the analysis to choose the significant MSPs and their corresponded GEMs. We then applied individual-level constraint-based modeling of these MSPs to understand the contribution of each species to the overall metabolic changes. In addition, we performed personalized community modeling together with personalized reaction abundance analysis. We also investigated the plasma metabolite association from a separate cohort of Swedish patients to evaluate the modeling predictions.

**Table 1:** Overview of metabolic disease cohorts per cohort

Cohort	Geographical region	Number of case samples	Number of control samples	Accession codes	Sequencing platform
T2D cohort 1	China	71	192	SRA045646 SRA050230	Illumina GAIIx and HiSeq 2000
T2D cohort 2	Sweden	93	39	ERP002469	Illumina HiSeq2000
Obesity Cohort 1	Denmark	71	89	ERA000116	Illumina
Obesity Cohort 2	Denmark	207	174	ERP003612	Illumina
ACVD cohort 1	China	214	171	ERP023788	Illumina HiSeq2000
ACVD cohort 2	Sweden	5	10	SRA059451	Illumina HiSeq2000

**Table 2:** Metadata on metabolic disease cohorts per disease

	Total	Female	Male	Age average	BMI average
T2D case	271	95	59	59.16	25.60
T2D control	231	77	66	47.36	23.49
Obesity case	278	126	124	56.79	33.55
Obesity control	263	107	105	56.12	23.49
ACVD case	219	33	79	62.35	24.83
ACVD control	181	51	40	60.70	24.75

### Identifying common and unique metagenome species across the three metabolic disorders

Phyla level analysis of the MSP profiles between patient and control groups in all three disorders showed Bacteroidetes and Firmicutes had consistent significant differences (FDR <0.01) (Figure S1). Comparing obese samples to their matched controls showed an increase in the abundance of Bacteroidetes, and decrease in the abundance of Melainabacteria*,* Verrucomicrobia, Tenericutes, Synergistetes, and Firmicutes phyla. In contrast, we found that the abundance of Bacteroidetes was decreased in patients with T2D and ACVD while other phyla, including Actinobacteria and Firmicutes, were increased.

**Figure 1:**
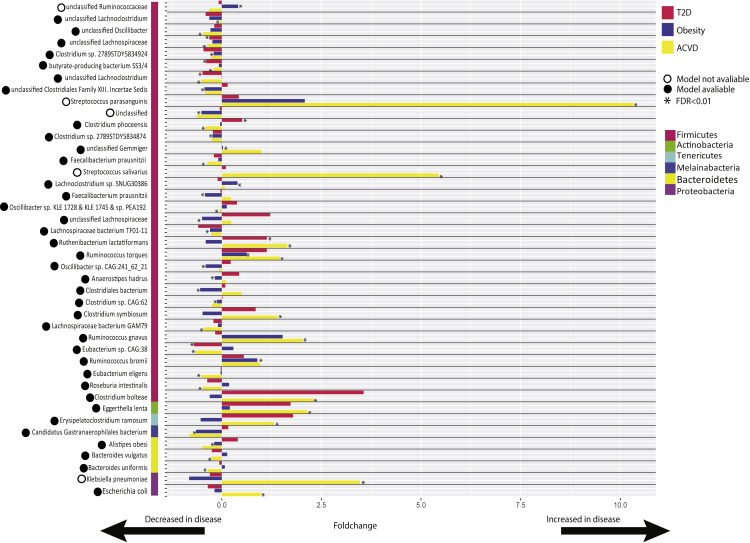
A schematic representation of the 42 unique and significant MSPs For each bacterial species, the red bar shows represent the enrichment in T2D, the blue bar represents the enrichment in obesity and the yellow bar represents the enrichment in ACVD. Fold-change>0 shows the species is increased in disease, fold-change <0 shows the species is decreased in disease. An asterisk (∗) next to a bar indicates the bacterial species was statistically significant in that disease when compared to the matched controls. A filled-in black circle next to the species name indicates a genome-scale metabolic model was available for that bacterial species, an empty circle shows there is no model available.

For a deeper insight at the species level, we identified significant MSPs within patients from all diseases (FDR <0.01) (Tables S1, S2, and S3). The highest abundance of enriched MSPs (fold-change) was in ACVD (65%), while there were 44 and 49% disease-enriched MSPs in obesity and T2D, respectively (Figure S2). We identified 202 statistically different (FDR<0.01) MSPs between patient and control groups in all cohorts; 139 significant MSPs in Obesity, 12 significant MSPs in T2D and 51 significant MSPs in ACVD. To evaluate the median abundance of the MSPs for the patients and matched controls, MSPs were selected based on having different medians between the two groups (17 MSPs for obesity, 6 for T2D, and 25 for ACVD). Grouping these together and removing any duplicated MSPs yielded 42 unique significant MSPs (Figure 1) (Figure S3, Table S4), with 33 MSPs out of the selected 42 from the phylum Firmicutes. The chosen 42 MSPs were not all significant in each of the 3 disorders. To determine if each MSP was enriched in the disease cases, we only considered the disease in which the MSP showed significance. This gave 15 disease-enriched MSPs and 26 control-enriched MSPs. Out of the six MSPs significant in ACVD and T2D, four were depleted in both diseases and one was enriched while *Clostridium phoceensis* was not consistent across the two diseases, being decreased in patients with ACVD but increased in patients with T2D.

### Genome-scale metabolic modeling showed changes in short-chain fatty acids and amino acid metabolism in metabolic diseases

**Figure 2:**
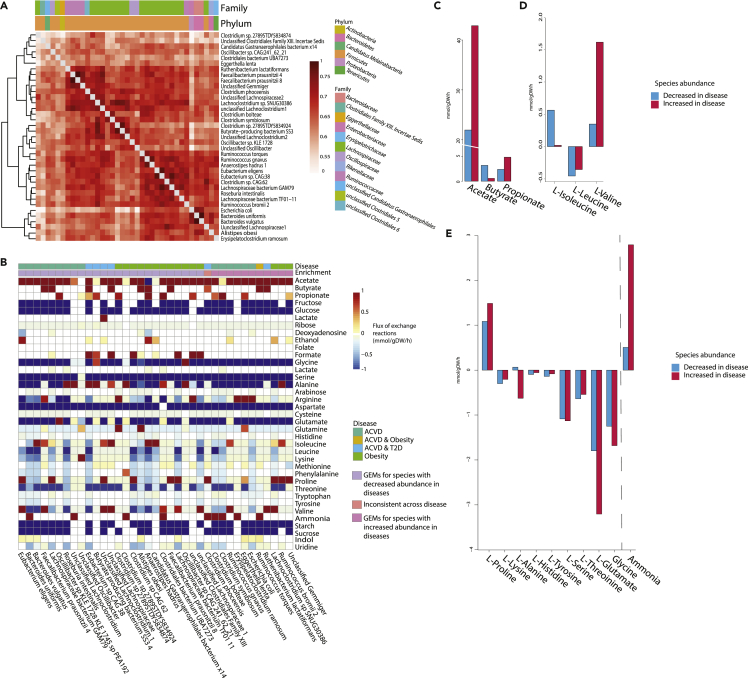
An overview of the individual genome-scale models and their individual fluxes (A) Jaccard index of the models, showing similarity between the different species based on reactions. Distances range 0-1 a score of one represents two models which are identical, a score of 0 represents two models which have no overlapping reactions. (B) Heatmap showing the flux for different metabolites of the models constrained by a high fiber omnivore diet, with the objective function to optimize biomass, using COBRA Toolbox. For visualization, flux was not shown for greater or lesser than +/−1. (C) The average flux from the models which were increased in disease (pink) compared to the average flux from the models decreased in disease (blue) for SCFAs. (D) The average flux from the models which were increased in disease (pink) compared to the average flux from the models decreased in disease (blue) for BCAAs. (E) The average flux from the models increased in disease (pink) compared to the average flux from the models decreased in disease (blue) for metabolites that show a difference between the average flux amounts. For C-E negative flux implies the metabolite is up taken by the model, positive flux implies the model secretes this metabolite.

In order to investigate the microbe-microbe interactions, we applied 37 functional GEMs for the MSPs associated with pathology. 11 models represented MSPs increased in ACVD, T2D, and obesity while 26 models represented MSPs decreased in all three disorders. A further model represented the inconsistent species *C. phoceensis* identified above. The selected models contained on average 1,717 reactions, 1,684 metabolites, and 1,022 genes with an average growth rate of 0.89 h^−1^ (Figure S4). The majority of the selected GEMs belonged to Lachnospiraceae and Ruminococcaceae families with 19 and 5 models, respectively. Furthermore, we showed that the models are metabolically distinct as determined by their Jaccard index, the average Jaccard index was equal to 0.637 (Figure 2A, Table S5). The models were assessed by constraining exchange reactions with a high fiber omnivorous diet as an input and setting biomass as the objective function (Bidkhori et al., 2021). We clustered the flux predictions from the models based primarily on the models’ enrichment in diseased patients and secondly on which disease the MSP was significantly enriched/depleted in (Figure 2B). The metabolism of sugars and amino acids was predicted in these models. Using sucrose, starch, fructose, and glucose which are available from the high fiber diet as inputs, models showed they produce indole, ammonia, and amino acids. In the species-level simulations, SCFAs were also produced; 35 models secreted acetate, 10 secreted butyrate, and 12 secreted propionate (Table S6). After comparing the fluxes in individual GEMs, we compared the average flux across those MSPs increased in disorder versus decreased. Average flux based on microbiome compositional changes in diseases showed the flux exchange of key metabolites such as SCFAs and amino acids was altered. Acetate and propionate flux were increased in species with higher abundances in patients from all disease cohorts (Figure 2C). The predicted flux of butyrate showed lower amounts being produced by species increased in disease. There were also notable differences between the branch chain amino acid (BCAA) flux profiles, with average valine production higher than those MSPs increased in disease. Finally, there were increases in the average production of ammonia and proline, as well as increased consumption of glutamate, glycine, alanine, and serine by those models representing species enriched in disease. (Figure 2D).

### Enrichment of tartrate metabolism among the three metabolic disorders

**Figure 3:**
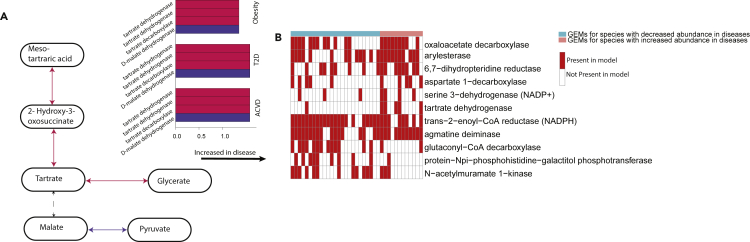
The presence of the glyoxylate and dicarboxylate metabolism in reaction analysis and the individual models (A) A pathway showing four of the reactions upregulated in the three diseases. Red arrows indicate reactions from the glyoxylate and dicarboxylate metabolism, purple arrows indicate reactions from the Butanoate metabolism. On the right, the bar-plot shows the enrichment of these four reactions in each disease. (B) Reaction presence in the models. Reactions were only considered if they were significant (Benjamini-Hochberg, Q < 0.01) in every disease and there was over 20% difference in the abundance between the presence of the increased MSPs and decreased MSPs. Red shows the reaction is present in the model, blank white shows the reaction is absent.

To further understand the functional behavior of the microbiome, we performed reaction abundance analysis for each individual using the MIGRENE Toolbox (Bidkhori et al., 2021) (Tables S7, S8, and S9, STAR Methods). A reaction abundance matrix was constructed for all samples, based on the MSP abundance table and the reactions present within the GEMs. Statistical analysis was then performed based on comparing the diseased cohorts to their matched controls. There were in total 820 significant reactions in obesity, 939 reactions in T2D, and 1,105 reactions in ACVD, compared to their healthy cohorts (FDR <0.01). From this, there were 214 unique reactions that were significant and present in all three diseases. We mapped the 214 significant reactions to KEGG orthologs (KOs). This resulted in 86 significant KOs (FDR <0.01) (Figure S5, Table S10). However, only five of these reactions from two different KOs were enriched consistently across all three diseases. To define the molecular functionality of these reactions, we mapped the reactions to the Kyoto Encyclopedia of Genes and Genomes database (KEGG) (Kanehisa et al., 2017) metabolic pathways (Figure 3A). From the five enriched reactions, two were involved in tartrate dehydrogenase and one with tartrate decarboxylase. These reactions are from the glyoxylate and dicarboxylate metabolic pathways.

As the reaction abundance analysis looks at the reactions in all models and is calculated based on the abundance of the corresponding MSPs consequently the reactions highlighted will be present in the 37 GEMs we used in the individual modeling. The corresponding 86 KOs from the reactions were present across the 37 GEMs, from this six reactions had higher presence in the GEMs for species enriched in disease (Figure 3B). These six reactions included tartrate dehydrogenase.

### Microbial community modeling shows decreased production of acetate and butyrate in patients with T2D

Following the analysis of individual GEMS and their potential contribution to metabolism in the gut microbiome, it is important to look at the microbial community as an integrated system. Hence, we developed 147 community models for each individual based on their species abundance for the two Swedish cohorts (93 T2D case, 36 T2D control, 5 ACVD case, and 10 ACVD control), using the MIGRENE Toolbox (Bidkhori et al., 2021). We ran simulations on these community models and implemented an optimization for the total biomass of the community model (Method). This was maximized such that the biomass of individual models within the community was maximized. The community models were constrained with a high fiber omnivorous diet, which only limited the uptake exchange reactions in the models. The average biomass production was 1.64 mmol/gDW/hr for the community models.

**Figure 4:**
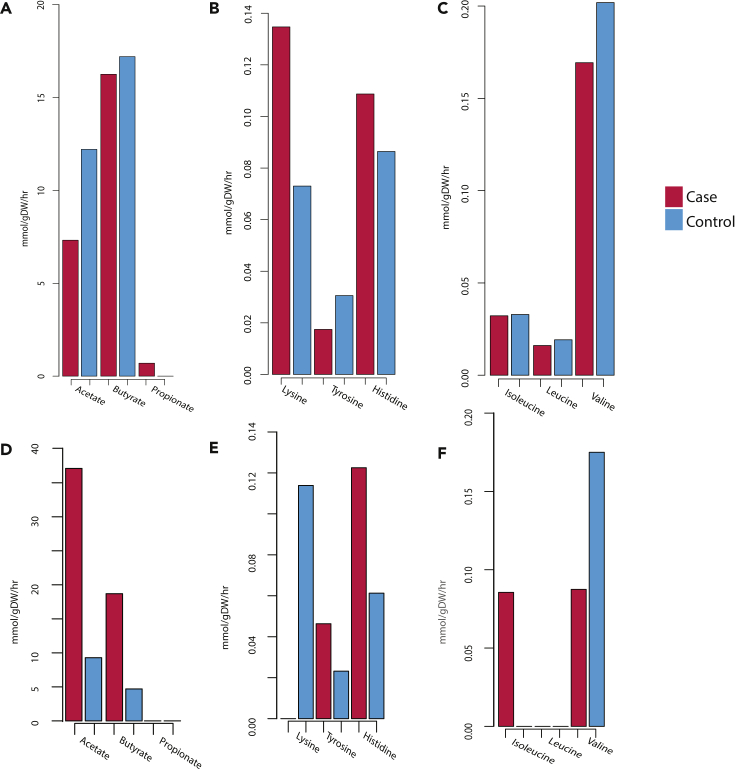
The average flux produced from community models (A) The average flux for SCFAs from personalized community models for patients with Swedish T2D (pink) compared to the average flux for SCFAs from personalized community models for matched controls (blue). (B) The average flux for AAs lysine, tyrosine, and histidine from personalized community models for patients with Swedish T2D (pink) compared to the average flux for AAs from personalized community models for matched controls (blue). (C) The average flux for BCAAs from personalized community models for patients with Swedish T2D (pink) compared to the average flux for BCAAs from personalized community models for matched controls (blue). (D) The average flux for SCFAs from personalized community models for patients with Swedish ACVD (pink) compared to the average flux for SCFAs from personalized community models for matched controls (blue). (E) The average flux for AAs lysine, tyrosine, and histidine from personalized community models for patients with Swedish ACVD (pink) compared to the average flux for AAs from personalized community models for matched controls (blue). (F) The average flux for BCAAs from personalized community models for patients with Swedish ACVD (pink) compared to the average flux for BCAAs from personalized community models for matched controls (blue).

The community models for individuals in the Swedish T2D cohort showed a common theme of lower production of key metabolites in the T2D communities. We observed on average the T2D community models predicted lower secretion of acetate and butyrate (Figure 4A), BCAAs (Figure 4B) and lysine, tyrosine, and histidine (Figure 4C) when compared to the average production flux from the control communities.

The community modeling for individuals in ACVD cohort predicted higher production of the butyrate and acetate, and the tyrosine and histidine in the diseased models (Figures 4D–4F), while the valine and the lysine were predicted with higher production in the control community models.

These findings are relevant as they highlight how the community behaves differently from the individual models. For example, the production of acetate which was shown to be highly secreted by the individual models increased in disease now shows lower production from the T2D community models when compared to the control community models. Also, in the ACVD community models, we observed the BCAA production differs from that predicted in the individual modeling.

### Bacterial tartrate metabolism showed a significant association with host plasma metabolites

To evaluate the importance of the microbiome tartrate and glyoxylate cycle in obesity, we investigated the plasma level of metabolites and gut metagenome from publicly available data on the longitudinal Swedish wellness study (Olsson et al., 2022; Tebani et al., 2020). Plasma metabolite profile allows us to link the gut microbial metabolism to the host physiology. This study used 101 healthy subjects with no clinically significant health complications ranging between 50 and 65 years of age. In this study stool and plasma samples have been taken from the patients. Previously, we performed the reaction abundance analysis on the gut microbiome of these individuals (Bidkhori et al., 2021).

We performed association analysis between the reaction abundance profile and metabolomics using multivariate association analysis. This metabolic analysis showed the association between reactions from the glyoxylate and dicarboxylate pathway and plasma metabolites in obese individuals of this cohort compared to non-obese ones. We used plasma metabolite profiles and BMI to find associations between the abundance of glyoxylate and dicarboxylate pathways in the gut using a multivariate random-effects model (MVEM). Focusing on reactions relating to glyoxylate metabolism we found associations between 14 reactions and 45 plasma metabolites belonging to carnitines, fatty acids and lipids, amino acids, steroids, and organic acids. (FDR <0.01, Figure S6, Table S11).

Malate dehydrogenase [NAD(P)+] in particular had five positive associations with proline, ketoleucine, histidine, glutamate, and alanine. Other reactions which were positively associated with alanine plasma levels were tartrate decarboxylase and tartrate dehydratase along with four other enzymes (Figure S7). Other amino acids which showed positive associations reactions were proline which had five positive associations and tyrosine which showed four. Whilst the amino acids histidine, tryptophan and ornithine all showed multiple negative associations with reactions within the glyoxylate metabolism.

**Figure 5:**
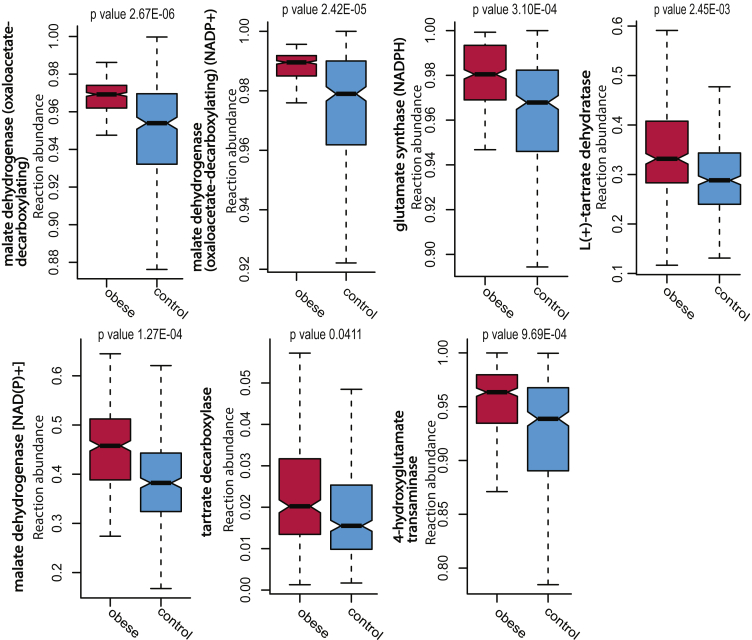
Comparison of reactions from the glyoxylate and dicarboxylate metabolism in Swedish wellness cohort Boxplots showing reaction abundance in the Swedish wellness cohort comparing those with BMI>30 (pink) and those with BMI <30 (blue). All reactions, except for tartrate decarboxylase, show significance (P-value<0.01).

To investigate if these microbial reactions were also associated with BMI, we split the Swedish cohort by individual patients’ BMI and compared the two subgroups. We ran statistical tests (Wilcoxon rank-sum test) to ascertain if the reactions were correlated with one group or the other. Of the 14 reactions, 7 showed a significant positive association with BMI, including tartrate dehydratase, tartrate decarboxylase, and malate dehydrogenase. 4 reactions (malate dehydrogenase (oxaloacetate-decarboxylating), malate dehydrogenase (oxaloacetate-decarboxylating) (NADP+), malate dehydrogenase [NAD(P)+], tartrate decarboxylase) were significantly increased in abundance in samples from obese participants compared to other non-obese participants (P-value < 0.05 Wilcoxon rank-sum test, Figure 5, Table S12). The increase of these reactions in the obese Swedish subjects validates the discovery of the enrichment of the bacterial glyoxylate and dicarboxylate metabolism in obese cohorts.

## Discussion

This study was undertaken to gain deeper understanding of gut microbiome dysbiosis and the role of bacteria in metabolic disorders. Our increasing understanding of the interactions between the gut microbiome and its host during disease is poised to reveal the mechanistic role of the microbiome and its collective metabolism on human health and disease pathophysiology. Metabolic diseases have all previously been linked to changes in the gut microbiome, but to date, they have not been looked at in an integrative fashion. This study demonstrates differences in three metabolic diseases when compared to healthy controls and highlights the link between the diseases.

Metagenomic analysis identified the bacteria present during dysbiosis, including highly prominent species from the Melainabacteria*,* Verrucomicrobia, Tenericutes, Synergistetes, and Firmicutes phyla. Our results showed *Clostridium bolteae, Clostridium symbiosum, Eggerthella lenta,* and *Escherichia coli* were increased in T2D, and these species have been shown to be increased in T2D before (Karlsson et al., 2013a; Qin et al., 2012b). *E. lenta* and *E. coli* have also been shown to be increased in ACVD before (Jie et al., 2017). *Ruminococcus gnavus*, *Ruminococcus torques,* and *Klebsiella pneumoniae* were also noted in our results as increased in ACVD and obesity, which have also previously been noted to be increased in ACVD and obesity (Jie et al., 2017; Le Chatelier et al., 2013b). Although *Eubacterium eligens, Clostridiales bacterium, Roseburia intestinalis,* and *Faecalibacterium prausnitzii* were all depleted in our results and have been shown to also be decreased in metabolic diseases before (Karlsson et al., 2013a; Qin et al., 2012b).

Further analysis using GEMs predicted phenotypic behavior - in particular, the substrate uptake and secretion profiles for SCFAs and amino acids. A large proportion of the models were from Lachnospiraceae, a genus whose members are known to ferment polysaccharides to SCFAs (Boutard et al., 2014). We further explored the bacterial behavior within an ecosystem by constructing personalized community models. These community models showed notable differences in the production of SCFAs and AAs, in particular BCAAs.

Based on individual model simulations, we predicted an increase in acetate and propionate production from species that were increased in the microbiome of patients of metabolic diseases. Acetate has been closely linked to inflammasome activation before, seeming to have a reducing effect on the inflammation (Xu et al., 2019). Propionate has also shown anti-inflammatory effects, suppressing NF-κB reporter activity (Tedelind et al., 2007). Elevated levels of BCAAs have been observed in patients with obese and T2D (Sikalidis and Maykish, 2020) and they have become biomarkers of insulin resistance helping predict the development of T2D (Zhu and Goodarzi, 2020). In addition, individual modeling showed an increase in glutamate consumption in species enriched in disease. Glutamate can induce obesity when administered in rodents, as well as has a positive correlation with obesity in Chinese adults (Lee et al., 2017). This individual modeling, therefore, shows that the metabolome profile of MSPs associated with these metabolic diseases is associated with the inflammation and physiology present in them. Here, we have shown how the gut microbiome, inflammation, and metabolic disease could be linked via these metabolites.

Reaction abundance analysis gave a deeper understanding of the mechanisms behind these results. Consistently throughout the three explored diseases, we found increased abundance of reactions in glyoxylate and dicarboxylate metabolism, in particular reactions surrounding tartrate. Tartrate is usually found in foods such as grapes and when consumed, it feeds into metabolism via the TCA cycle or is converted to glycerate (Kohn and Jakoby, 1968). Increased plasma levels of glycerate are positively correlated with T2D (Reddivari et al., 2017). Only 20% of tartrate ingested in food is eliminated in urine meaning the remaining ingested tartrate is potentially consumed by the microbes in the gut (Finkle, 1933). Although it is possible for human tissues to metabolize tartrate, the intestinal bacteria metabolize the bulk of it (Chadwick et al., 1978) highlighting the close connection between tartrate and the gut microbiome. Enteric bacteria can use both malate and tartrate (C_4_-dicaroboxylates) for anaerobic growth (Kim and Unden, 2007). Furthermore, tartrate can feed into proline and arginine metabolism, potentially causing increased production of these metabolites. Increased plasma levels of amino acids including proline and arginine have been seen in disease and could potentially lead to exacerbating disease (Proffitt et al., 2020).

Glyoxylate and dicarboxylate metabolism has previously been linked to atherosclerosis (Chen et al., 2018) and to obesity (Beaumont et al., 2016). The glyoxylate cycle can metabolize fatty acids to glucose which can then contribute to insulin resistance (Song, 2000). As tartrate (a metabolite within the glyoxylate and dicarboxylate metabolic pathways) has been associated with glutamate, this metabolite could be the missing link in the causative effect of glutamate on increased BMI. The increased production of acetate by the GEMs seen during metabolic disease could be linked to the glyoxylate and dicarboxylate metabolism as these pathways catabolize acetate and amino acids for energy production in the microbe (Maniscalco et al., 2017). There was also an increase in the arginine and proline metabolism. This increase also has a connection to the increased consumption of glutamate, which is necessary for the synthesis of arginine and proline (Cappelletti et al., 2018).

The consistency in the present results highlighting tartrate metabolism provide strong support for our models correctly predicting the phenotypes observed in the three studied metabolic diseases. The finding that reactions involved with tartrate along with functional findings from the plasma metabolite data in the separate study from Swedish cohorts provides strong support for this hypothesis. These findings validated the predictions of increased production of acetate, proline, and arginine from the metabolic modeling. It is also known that malate dehydrogenase [NAD(P)+] and tartrate dehydratase both produce oxaloacetate while malate dehydrogenase (oxaloacetate decarboxylate) produces pyruvate (Kim et al., 2007). Both oxaloacetate and pyruvate are used in the amino acid synthesis where aspartate, alanine, asparagine, methionine, lysine, and threonine are synthesized (Marty-Teysset et al., 1996), further validating our findings.

From these findings, we would hypothesis that an increased abundance of tartrate metabolism is not beneficial for health. This is based on the reactions showing an increase across all three of our diseases, also the fact that the gut microbiome metabolizes this metabolite. Tartrate is fermented in the colon to SCFAs (EFSA Panel on Food Additives and Flavourings et al., 2020), we saw acetate had a higher production from the individual modeling which correlates with tartrate metabolism producing SCFAs. Increased acetate can affect host metabolism by causing increased pancreatic β-cell activity, glucose-stimulated insulin secretion (GSIS), hyperphagia, and obesity (Bose et al., 2019). To take this work furthermore, computational protein or gene expression analysis could ensure the presence of the tartrate pathway within the bacterial species we have seen to be enriched in diseases. Additionally, more metabolomic analysis of stool and plasma from patients with T2D or ACVD could further validate these findings. Taking this idea further microbial culture metabolomics could be analyzed for comparison with the patient metabolomics to confirm the hypothesis from patient metabolomics.

This analysis can be used to unravel the association between impaired gut microbiomes and metabolic disorders. We have identified tartrate metabolism in the microbiome as a significant pathway with high potential to impact on each of these metabolic syndromes. As well as this giving a potential new biomarker, it also suggests potential novel intervention targets for disease. Further research into the metabolism of tartrate in the gut environment is needed to understand the direct impact this compound has on gut microbes and host health. It is clear that the gut microbiome is a key factor in maintaining health and our highlighted novel areas for research give areas to focus on for both disease understanding and biomarker discovery for obesity and its co-morbidities.

### Limitations of the study

There are a number of limitations to our study. Firstly, the cohorts chosen to represent the metabolic diseases are from different geographical regions which will have its own impact and implications on the gut microbiome profile. There are also limitations within the modeling of the bacteria. There are several bacterial species in the gut (Almeida et al., 2019), while we only had 1, 333 bacterial species-specific models available from the MIGRENE toolbox. Another limitation within the modeling was the computational power limits which restricted the number of models within the community modeling to 10 GEMs per community. Clearly, if this could have been increased the community models would have been a better representation of the ecosystem within the gut microbiome.

## STAR★Methods

### Key resources table

**Table:** 

REAGENT or RESOURCE	SOURCE	IDENTIFIER
**Software and algorithms**		

Meteor	(N. Pons et al., 2010, JOBIM, conference); (Wen et al., 2017)	https://www.academia.edu/14061278/METEOR_a_plateform_for_quantitative_metagenomic_profiling_of_complex_ecosystems
Trimmomatic	(Bolger et al., 2014)	http://www.usadellab.org/cms/?page=trimmomatic
Bowtie2	(Langmead and Salzberg, 2012)	http://bowtie-bio.sourceforge.net/bowtie2/index.shtml
Integrated Gut Catalog version 2 (IGC2)	(Wen et al., 2017)	https://data.inrae.fr/dataset.xhtml?persistentId=doi:10.15454/QVCYRB
MetaOMineR	(Le Chatelier et al., 2013a)	https://cran.r-project.org/web/packages/momr/index.html
KEGG	(Kanehisa et al., 2017)	https://www.genome.jp/kegg/
KBase	(Arkin et al., 2018)	https://www.kbase.us
COnstraint-Based Reconstruction and Analysis (COBRA) Toolbox (v2.0)	(Schellenberger et al., 2011)	https://opencobra.github.io/cobratoolbox/stable/index.html
MIGRENE Toolbox	(Bidkhori et al., 2021)	https://github.com/sysbiomelab/MIGRENE

### Resource availability

#### Lead contact

Further information and requests for resources and reagents should be directed to and will be fulfilled by Saeed Shoaie (saeed.shoaie@kcl.ac.uk).

#### Materials availability

This study did not generate new reagents.

### Experimental model and subject details

All the data used in this study has been published before.

### Method details

#### Datasets, processing and metagenomic downstream analysis

Datasets were obtained from six different studies of the human gut microbiome in metabolic diseases. Samples were discarded if the reading depth was below 10 million reads or if the there was no metadata available for the sample. The T2D studies consisted of 370 samples from a Chinese population, IDs SRA045646 and SRA050230, and 132 samples from a Swedish population, ID ERP002469. ACVD studies included 385 Chinese samples, ID ERP023788 and 15 Swedish samples, ID SRA05945. The obesity studies were both from Denmark, with 541 samples from ID ERA000116 and ID ERP003612. All datasets are available in the European Bioinformatic Institute (EBI) and Sequence Read Archive databases. All data was produced from different next generation sequencing (NGS) platforms. We used a quantitative metagenomic profiling software METEOR (Pons et al., 2010) for quality control, trimming and mapping to create gene count tables. To reduce variability, downsizing was done on the gene count tables (threshold of 10 million). Following this, MetaOMineR (Le Chatelier et al., 2013b) was used for normalization of gene counts for species abundance (herein MSP).

#### Reconstruction of individual microbial metabolic models

The models were constructed based on the Kegg Ontology annotation of the genes in the gut catalogue. The KBase reference model was used for mapping the KOs of the genes to the reactions (Price et al., 2018) using function “microbiomeGEMgeneration” in MIGRENE toolbox. The reactions were scored based in the information for each bacterium from the catalogue. Taxonomy profiles and the KBase reference model were used for gap filling in order to make the models functional using MIGRENE toolbox. The models from the MIGRENE toolbox are known as the Metagenome species Assembled Genome-scale MetAbolic model (MAGMA) models. These models were created based on the gut microbial gene catalogue (Wen et al., 2017) and metagenomic species pan-genomes (MSPs). There are 1,333 functional MAGMA models, in this instance, a functional model means it is balanced for mass, energy and solution capacity. The models had an average completeness of 83.6 ± 22.5%, while the non-functional models had a completeness of 48.4 ± 35.6%. The non-functional models were not used in the analysis. The MIGRENE toolbox is a comprehensive platform to analyse the reactobiome, the microbiome composition, metagenome species and community models.

The functional models were constrained based on a high fibre omnivore diet under anaerobic conditions. The diet was obtained from MIGRENE toolbox and fitted to the selected models. The growth rate was measured for each model by steady state simulation COnstraint-Based Reconstruction and Analysis (COBRA) toolbox (Heirendt et al., 2018) in MATLAB and using the linear program solver gplk. Predictions were made based on flux balance analysis (FBA); models were constrained based on a high fibre omnivore diet (https://github.com/sysbiomelab/MIGRENE) with the objective function to optimize biomass. Model growth rates and exchange reaction flux were determined by FBA for selected significant MSPs (Data S1). The similarity between two models was calculated using the Jaccard distance. The distance was computed by D = 1–|Ri∩Rj|/|Ri∪Rj|, with R_i_ as the set of reactions from model i and R_j_ the set of reactions from model j. Hence, if D = 0, the models are identical, if D = 1, the models are completely dissimilar.

#### Metabolic reaction abundance analysis

Reaction abundance was calculated based on the absence or presence of reactions in the respective MSP models (Data S1). The reactions were multiplied by the MSP abundance, to personalize the reaction abundance to each sample, we summed the frequency the reaction repeated in the whole microbiome of the sample. Significantly different reaction between case and control were identified using the two-tailed Wilcoxon rank-sum test (P-value) and the false discovery rate by the Benjamini-Hochberg correction (FDR<0.01). Reactions of significance were mapped to KEGG orthologs (KOs) or Enzyme Nomemclature (EC numbers).

#### Community metabolic models based on disease, health status and geography

Personalised community models were reconstructed for all Swedish samples. Due to computational power limitations the models were made with 10 MSPs per community. The top-ranked 10 bacteria for each sample were chosen for the community models. The models were created using function “MakeCommunity” in MIGRENE toolbox (Data S1). The objective function was the biomass of the entire community.The resulting community model consisted of all reactions from the MSPs within the community, and all metabolites presents. Three new compartments were designed: 1) lumen, a compartment for all the bacteria in gut and their interactions to allow metabolites to be secreted and up taken by the bacteria. 2) A compartment including exchange reactions for food input and 3) and a compartment for another type of exchange reactions to remove metabolites from the system (to faeces/blood). FBA analysis was ran on the community models where the biomass of species were constrained based on the corresponding abundance in the community biomass as objective function. These simulations were run on COBRA toolbox.

### Quantification and statistical analysis

#### Microbial signatures of metabolic disease

All statistical analysis was done using R software. Different abundance of taxonomic signatures were tested using the two-tailed Wilcoxon rank-sum test in controls and patients. p values were adjusted for false discovery rate by the Benjamini-Hochberg correction (FDR). Significant MSPs were chosen based on 2 steps: (1) the MSP’s FDR score, FDR<0.01, (2) distinctly different median values in the patients and controls, one equal to 0 and one equal to a real positive number. We estimated the MSP enrichment or depletion in diseases by comparing the average abundance in case samples, and the average abundance in control samples per MSP. The output identified each MSP as either enriched in disease or enriched in control.

#### Longitudinal Swedish cohort samples metabolomics

The initial analysis for the associations between the plasma metabolite levels and reaction abundancies within the gut microbiome was done previously (Bidkhori et al., 2021), using available metabolomics and metagenomics (Olsson et al., 2022; Tebani et al., 2020). Here, the plasma and stool samples from the Swedish cohort were linked using the reaction abundance. The plasma samples gave circulating metabolite profile and the stool samples gave reaction abundance from the gut microbiome. Following this we ran multivariable association analysis on the data to find the corelation between the clinical metadata and microbial omics features. This analysis was done in R using packages *taRifx, Maaslin2* and *car.*

## Data Availability

All the metagenomics data used in this study have been publicly available in the European Bioinformatic Institute (EBI) and Sequence Read Archive databases. T2D studies were under the study accession SRA045646, SRA050230 and ERP002469. ACVD studies were under the study accession ERP023788 and SRA05945. The obesity studies were under the study accession ERA000116 and ERP003612. The Swedish wellness cohort metagenome data can be found under the study accession PRJEB38984. All the metabolic models used in this study are available at Database: https://www.microbiomeatlas.org/downloads.php.

## Supplemental information

Document S1. Figures S1–S7 and Data S1

Table S1. MSP analysis for obese cohorts, related to STAR Methods Obesity MSP analysis including species name, MSP ID, the P value and P adjusted value from Wilcoxon test, the mean abundance of the msps from the patients (mean1) and controls (mean2), and the fold-change.

Table S2. MSP analysis for T2D cohorts, Related to STAR Methods T2D MSP analysis including species name, MSP ID, the P value and P adjusted value from Wilcoxon test, the mean abundance of the msps from the patients (mean1) and controls (mean2), and the fold-change.

Table S3. MSP analysis for ACVD cohorts, related to STAR Methods ACVD MSP analysis including species name, MSP ID, the P value and P adjusted value from Wilcoxon test, the mean abundance of the MSPs from the patients (mean1) and controls (mean2), and the fold-change.

Table S4. Overview of MSPs chosen for analysis, related to Figure 1 MSPs chosen for analysis, with the fold-change of the MSP, the median difference between patients and controls, the disease which the MSP is significant in, all taxonomy information, and if there is a model for the MSP.

Table S5. Jaccard index of models, Related to Figure 2 The Jaccard index value between all models FBA results for all metabolites and all models.

Table S6. FBA analysis results, Related to STAR Methods The fluxes for the exchange reactions from the individual models.

Table S7. Obesity reaction analysis including reactions KO ID reaction ID, the P value and P adjusted value from Wilcoxon test, the mean abundance of the reaction from the patients (mean1) and controls (mean2), and the fold-change.

Table S8. Reaction abundance analysis for T2D cohorts, related to STAR Methods Reaction abundance analysis for obesity cohorts, Related to STAR Methods – T2D reaction analysis including reactions KO ID, reaction ID, the P value and P adjusted value from Wilcoxon test, the mean abundance of the reaction from the patients (mean1) and controls (mean2), and the fold-change.

Table S9. Reaction abundance analysis for ACVD cohorts, Related to STAR Methods ACVD reaction analysis including reactions KO ID, reaction ID, the P value and P adjusted value from Wilcoxon test, the mean abundance of the reaction from the patients (mean1) and controls (mean2), and the fold-change.

Table S10. 86 Kos present in all GEMs, related to Figure 3 The 86 KOs which were present across all 37 GEMs modeled.

Table S11. Association between plasma metabolites and the tartrate and glyoxylate cycle reactions in the Swedish cohort, related to STAR Methods associations between plasma metabolites and the tartrate and glyoxylate cycle reactions, including reaction number, ec number, the P value, and P adjusted value from Wilcoxon test and names of the metabolites and reactions.

Table S12. Association between BMI and reactions in Swedish cohort, related to Figure 5 association between BMI and reactions in the longitudinal Swedish cohort, including, reaction number, ec number, P value from Wilcoxon test and feature name.
