# Supplementary material for: Genome-scale metabolic modelling of the human gut microbiome reveals changes in the glyoxylate and dicarboxylate metabolism in metabolic disorders
Source: iScience. 2022 Jun 2;25(7):104513. doi: 10.1016/j.isci.2022.104513 (PMC9213702; doi:10.1016/j.isci.2022.104513)
Supplement: Document S1. Figures S1–S7 and Data S1 [file mmc1.pdf]

iScience, Volume *25*

Supplemental information

Genome-scale metabolic modelling of the human gut microbiome reveals changes in the glyoxylate and dicarboxylate metabolism in metabolic disorders

Ceri Proffitt, Gholamreza Bidkhori, Sunjae Lee, Abdellah Tebani, Adil Mardinoglu, Mathias Uhlen, David L. Moyes, and Saeed Shoaie

Supplementary 1

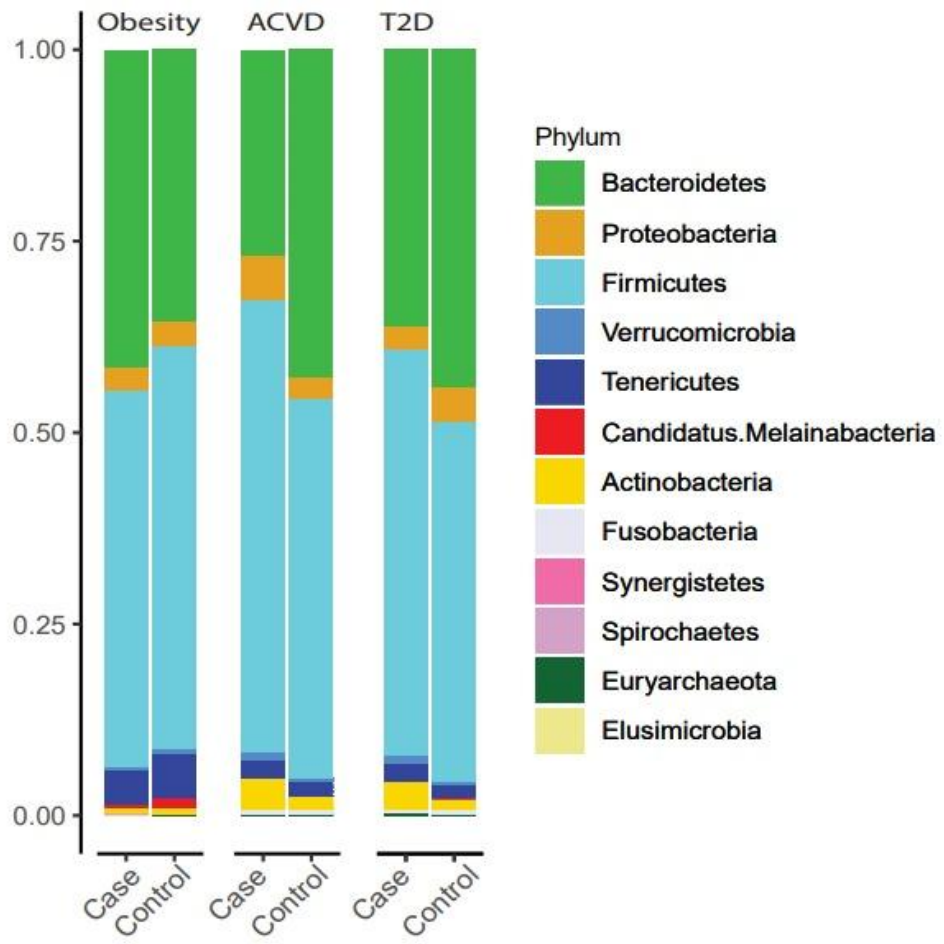

Supplementary figure 1. The relative abundancy of each phylum, Related to STAR Methods

The relative abundancy of each phylum, cases vs. controls, in all 3 diseases. The abundance of firmicutes and Bacteroidetes changes between case and control each time, differences can also be seen in proteobacteria, tenericutes and actinobacteria.

Supplementary 2

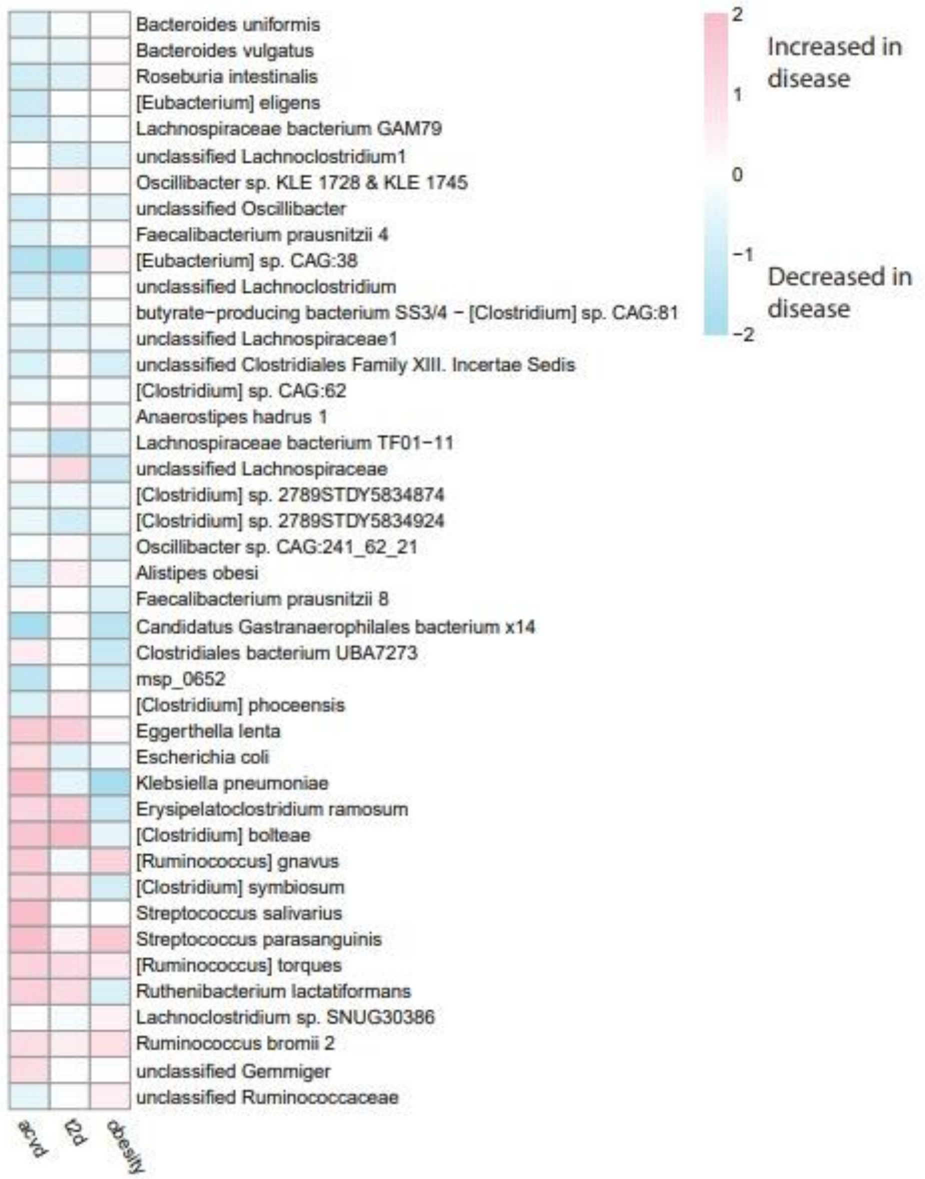

Supplementary figure 2. Enrichment of the 42 MSPs in each disease, Related to figure 1

The 42 MSPs chosen to study in detail in the paper and their enrichment in disease (log2(foldchange)). Pink shows the MSP is increased in the disease and blue shows the MSP is decreased in the disease. The enrichment is shown for ACVD, T2D and obesity, the differences in enrichment in the disease can be seen.

Supplementary 3

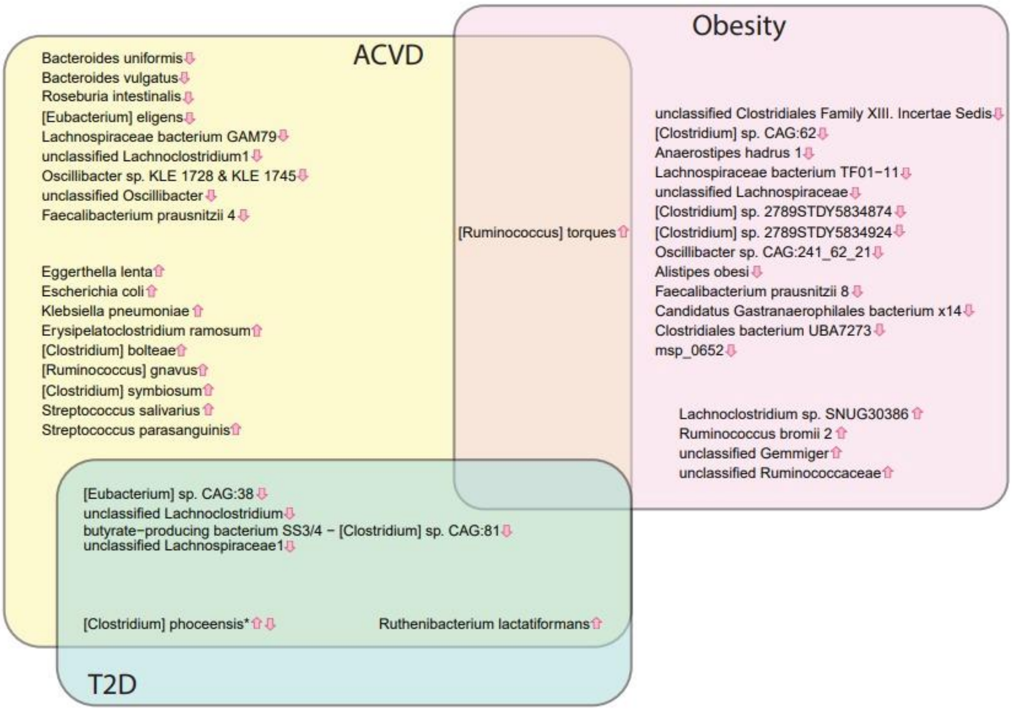

Supplementary figure 3. The 42 MSPs grouped by the disease, Related to figure 1

A venn diagram of the names of the 42 MSPs studied in detail, grouped by the diseases they are significant in. Arrows indicated the enrichment of that species seen in that disease. [Clostridium] phoceensis* is significant in T2D and ACVD, however is decreased in ACVD patients but increased in T2D patients.

Supplementary 4

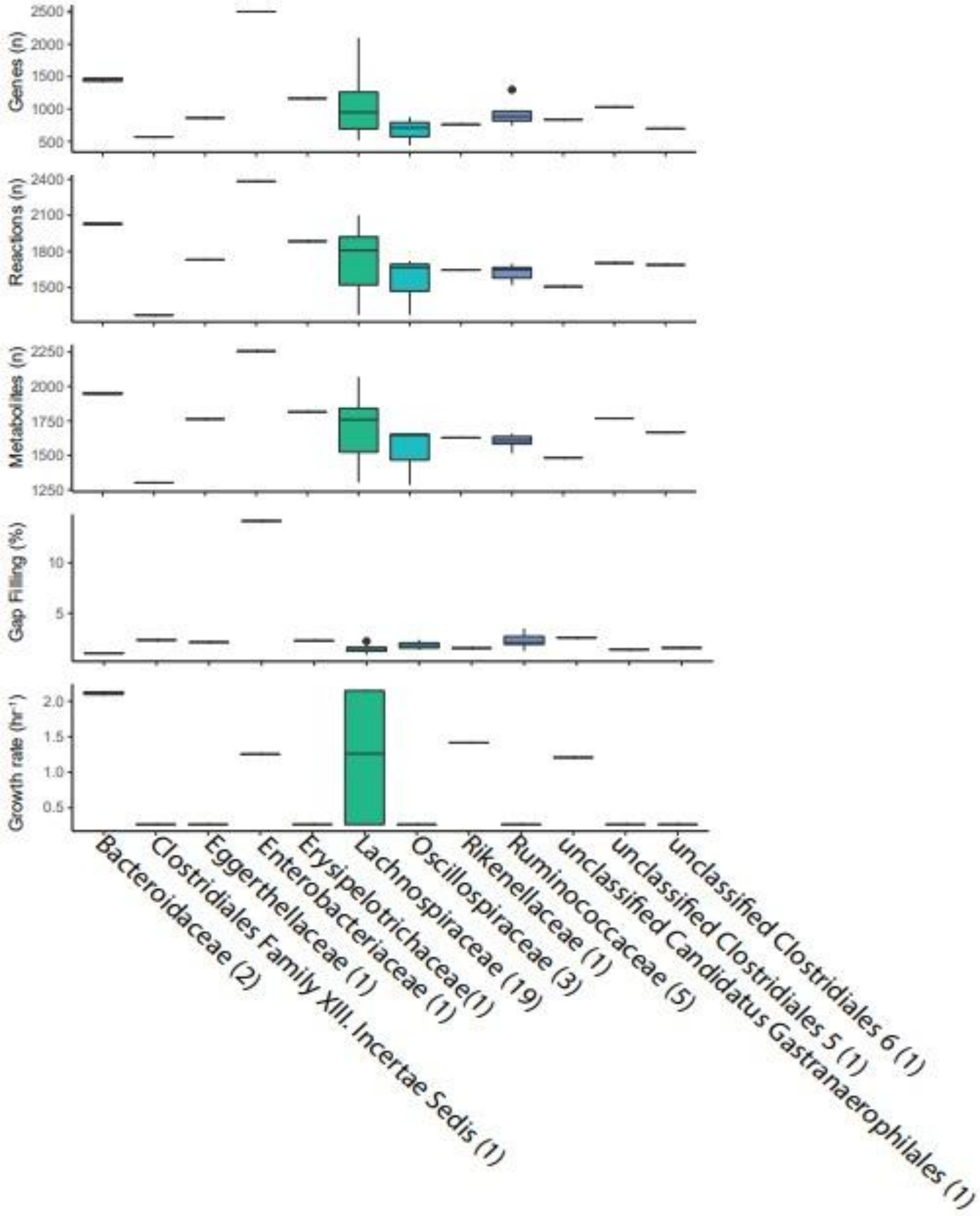

Supplementary figure 4. Overview of 37 functional models, Related to figure 2

Overview of 37 functional models, shown by family the models belong to and how many models belong to each family. The number of reactions, metabolites and genes present in the models are shown, also the growth rate of the models based on a high fibre omnivore diet and the percentage of gap filling done to make the models functional.

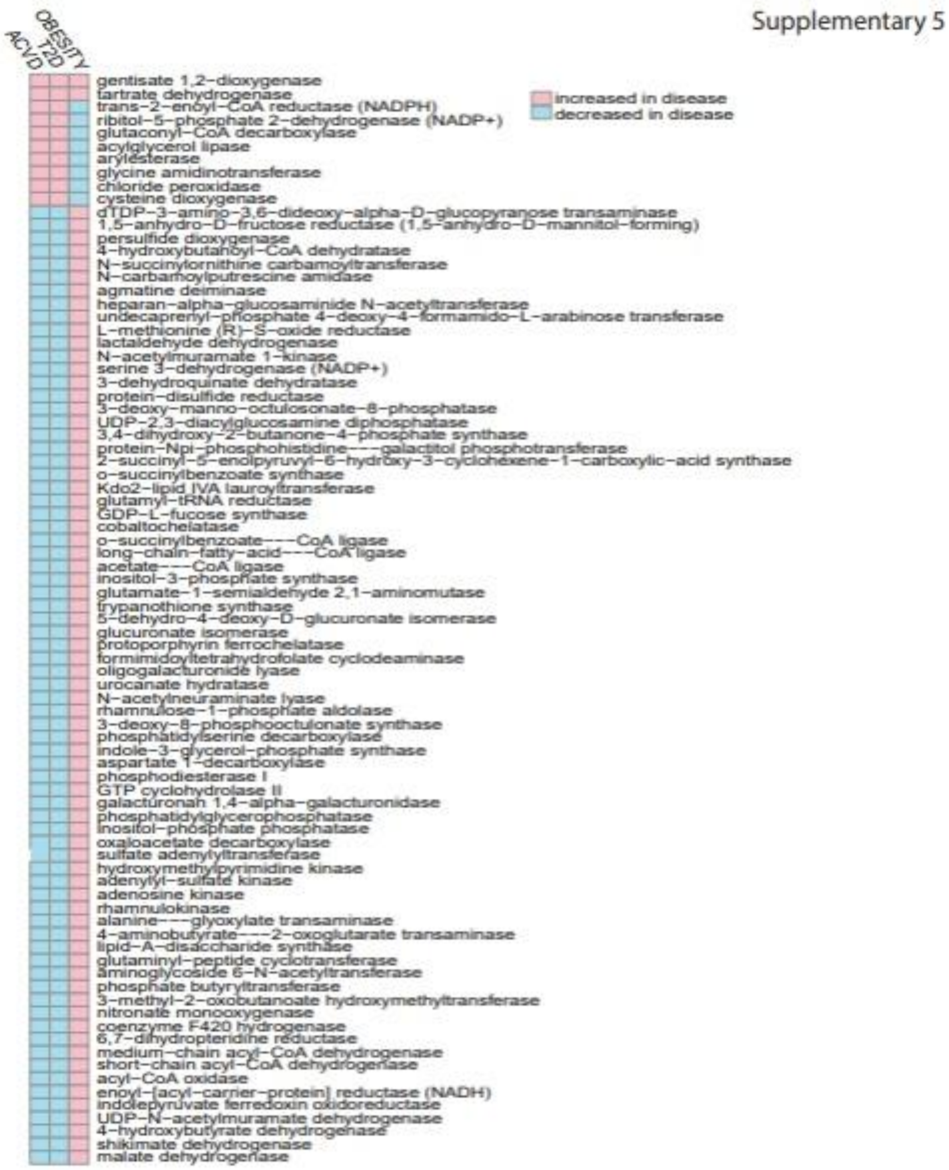

Supplementary figure 5, Enzymes related to significant reactions, Related to figure 3

The 83 enzymes (from KEGG) related to significant reactions which were significant in every disease (Benjamini–Hochberg, Q< 0.01), showing the enrichment of the enzyme in the diseases. Pink shows the enzyme is increased, blue shows the enzyme is decreased.

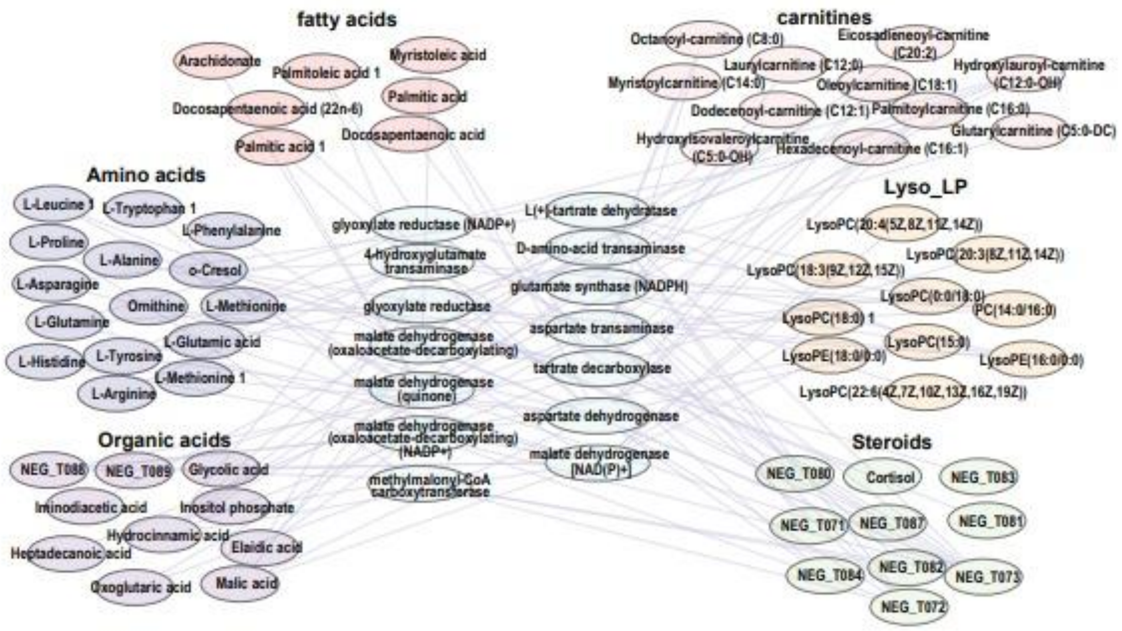

Supplementary figure 6 Network of associations between the tartrate and glyoxylate cycle and plasma metabolites, Related to figure 5

Network of associations between reactions from the tartrate and glyoxylate cycle and plasma metabolites including carnitines, lyso_LPs, steroids, fatty acids, amino acids and organic acids.

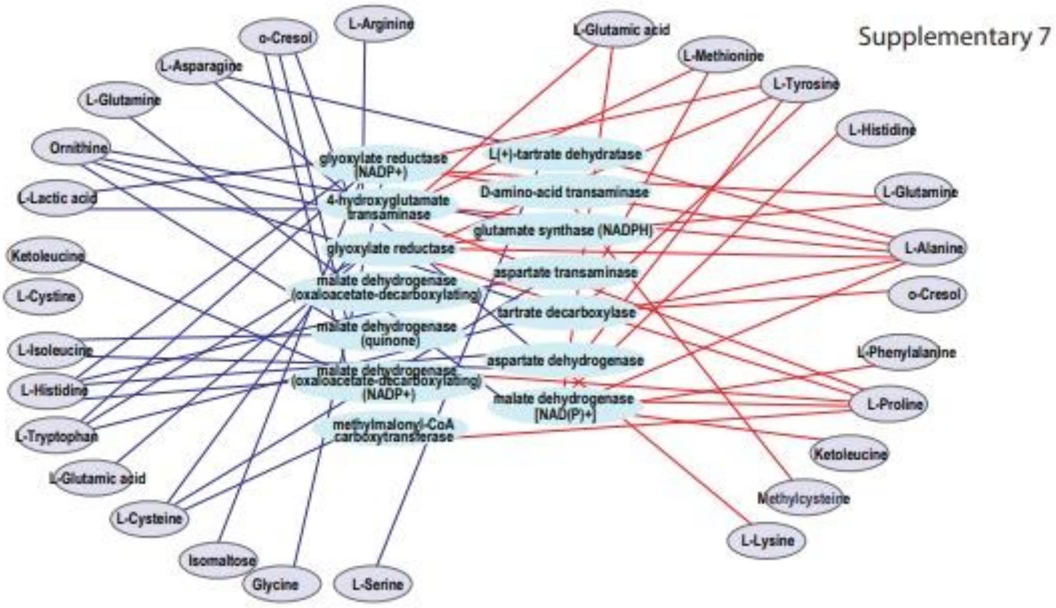

Supplementary figure 7. Network of associations between the tartrate and glyoxylate cycle and plasma amino acid levels, Related to figure 5

Network of associations between reactions from the tartrate and glyoxylate cycle and plasma amino acid levels; red connections show a positive association between the reaction and the metabolite and blue shows a negative association between the reaction and the metabolite. If these associations are positive (there is a positive correlation between the reaction in the gut and the plasma metabolite) or negative (a negative correlation).

Data S1. Pseudo code for matlab analysis , Related to STAR Methods

Pseudo code showing the input needed in matlab for FBA on individual models, the personalised reaction abundance analysis and creating the community models and running FBA on them.

```
addpath('cobratoolbox')
initCobraToolbox

%Individual FBA
load('model.mat');
load('diets.mat');


ExchangeRxns=model.rxns(find(~(cellfun('isempty',s
trfind(model.rxns,'Ex_'))))));
model.lb(ismember(model.rxns,ExchangeRxns))=0;
    for x =1:length(diets)
        model.lb(find(strcmp(model.rxns,
diets{x})))=-(diets.value(x,2));
    end
 model=changeObjective(model,"Biomass");
 output=optimizeCbModel(model)

 %reaction abundance
addpath('MIGRENEtoolbox')
ABUNDANCE='BacteriaAbundance.xlsx'  %File with
bacterial abundance per sample
[abundance,infoFile,~]=xlsread(ABUNDANCE);
modelList = infoFile(2:end,1);
sampleName=infoFile(1,2:end);
PathToModels.path= 'MSPmodels' ;
PathToModels.name='model';
[reactionRelativeAbun, rxnAbunPerSample]=
ReactionAbundanceGenerator(modelList,PathToModels,
abundance,sampleName);

 %makeing community  models
ABUNDANCE='BacteriaAbundance.xlsx' %File with
bacterial abundance per sample
[abundance,infoFile,~]=xlsread(ABUNDANCE);
modelList = infoFile(2:end,1);
```

```
sampleName=infoFile(1,2:end);
PathToModels.path='MSPmodels'
PathToModels.name='model'
PathToSave='communitymodel'

MakeCommunity(modelList,PathToModels,abundance,sam
pleName)

 %FBA on Community
load('communitymodel.mat'); %load community model
load('diets.mat');

diets=replace(diets.rxn, "Ex", "FoEx") %change
diet precursor to FoEx to match community model

ExchangeRxns=model.rxns(find(~(cellfun('isempty',s
trfind(model.rxns,'FoEx_')))));
model.lb(ismember(model.rxns,ExchangeRxns))=0;
    for x =1:length(diets)
        model.lb(find(strcmp(model.rxns,
diets{x})))=-(diets.value(x,2));
    end
 model=changeObjective(model,"BiomassAll");
 output=optimizeCbModel(model)
```
